# Ambidextrous Leadership and Employee Work Outcomes: A Paradox Theory Perspective

**DOI:** 10.3389/fpsyg.2020.01661

**Published:** 2020-09-10

**Authors:** Zhuopin Guo, Jiaqi Yan, Xiaoying Wang, Jie Zhen

**Affiliations:** ^1^School of Economics and Management, Tongji University, Shanghai, China; ^2^Business School, East China University of Political Science and Law, Shanghai, China

**Keywords:** ambidextrous leadership, leader–member exchange, job performance, creativity, congruence

## Abstract

Numerous studies have examined the influence of ambidextrous leadership on employee work outcomes, but few have explored the issue through the congruence or incongruence of two seemingly conflicting leadership styles. Based on paradox theory, we adopted polynomial regression and surface analysis methods to investigate the congruent/incongruent effects of loose and tight leadership techniques. In order to reduce common method bias, we used a two-wave design with a two-month time interval. By using two-wave surveys of 301 employees, this study posited that ambidextrous leadership congruence creates higher leader–member exchange quality, and that loose and tight leadership with a high strength plays a more positive role in enhancing LMX quality. This study demonstrated that LMX quality mediates the relationship between ambidextrous leadership congruence/incongruence and employee work outcomes (i.e., job performance and creativity).

## Introduction

In China, hotels and the wider hospitality industry face fierce competition due to the changes and challenges brought about by a growth of tourism in recent years (Richard, 2017). Employees in these hotels have conflicting work demands, which may cause dilemmas for managers and leaders. A new generation of employees (born after 1980) comprise the main workforce in contemporary Chinese hotels. This younger generation prefer a participative communication style and greater job autonomy (Zhu et al., 2015), which is in opposition to the expectations of older employees, who prefer specific instructions and consistency from their supervisors (Hou et al., 2018). Hotel managers experience difficulties when attempting to accommodate these opposing needs, as they tend to emphasize one behavior over the other. Paradoxical thinking, as discussed by Smith and Lewis (2011), is a way of tackling these contingent issues and satisfying opposing employee demands in dynamic and competitive business environments. Paradox is a state where two contradictory and independent elements coexist in one sphere (Putnam et al., 2016; Cunha and Putnam, 2019). Paradox theory shows that two seemingly conflicting elements may coexist harmoniously to form a totality (Cameron and Quinn, 1988). Therefore, we propose that leadership based on paradox theory, a ‘both–and’ strategy of leadership, may provide a fruitful way for managers to meet these varied needs whilst also sustaining long-term performance (Zhang Y. et al., 2015; Waldman and Bowen, 2016).

In terms of power, ambidextrous leadership comprises loose leadership and tight leadership (Sagie, 1996). The main concern in previous studies was the impact of ambidextrous leadership on organizational and individual levels (Masa’deh et al., 2016; Zacher et al., 2016). However, the simultaneous effect of these two types of leadership remains unclear. Prior research has considered one pole of ambidextrous leadership (i.e., loose leadership or tight leadership) on the assumption that one pole is more prominent than the other under transient situational factors (Li et al., 2018). In other words, tight control and autonomy are incompatible simultaneously, and leaders may choose one approach, depending on the situation (Pacheco and Webber, 2016; Vasilagos et al., 2017). However, the paradoxical ‘both–and’ strategy asserts that incompatible paradoxes can applied as integrated parts of a larger whole, and that loose–tight ambidextrous leadership may maintain long-term control by granting employees the discretion to bend rules (Cunha et al., 2019). Unfortunately, studies that explore the effect of loose–tight leadership remain limited, with little exploration of how the combination of two seemingly conflicting leadership styles impacts work outcomes.

Examining literature on paradoxical leadership and research on leader–member exchange (LMX), this study discusses congruence and/or incongruence and how these affect loose and tight leadership. First, we contribute to paradox theory by constructing an ambidextrous leadership congruence model, using loose leadership and tight leadership. Prior studies posit that two seemingly conflicting leadership styles are incompatible simultaneously when they work separately in accordance with leadership contingency (Clark et al., 2009; Wu et al., 2013). Paradox theory confirms that incompatible leadership can coexist in one situation. In this study, we discuss how two seemingly conflicting leadership styles can coexist in ambidextrous leadership. To study the above question, we construct a loose–tight ambidextrous leadership model and integrate loose leadership and tight leadership into four combinations according to their different strengths (i.e., high–high, low–low, low–high and high–low). We confirm the legitimacy of this paradox, before exploring how these two seemingly conflicting leadership styles influence employee work outcomes, and the inner mechanisms of this approach.

Our findings support the paradoxical/ambidextrous leadership (in)congruence model by Jia et al. (2018), who posit that paradoxical/ambidextrous leadership incongruence is a pivotal antecedent to follower behavior. This contributes to the boundary condition of the relationship between paradoxical/ambidextrous leadership incongruence and follower behavior. Although the model by Jia et al. (2018) is helpful, two important issues remain uncertain. On the one hand, Jia et al. (2018) explore the boundary condition, but fail to analyze the mediation mechanism between paradoxical/ambidextrous leadership (in)congruence and follower behavior. On the other hand, Jia et al. (2018) concentrate on followership behavior with little analysis of how paradoxical/ambidextrous leadership (in)congruence influences the work outcomes of employees. In response, this study examines how LMX quality mediates loose–tight ambidextrous leadership congruence in the job performance and creativity of subordinates.

Finally, this study extends LMX literature by examining loose–tight ambidextrous leadership as antecedents of LMX. Previous studies have discussed the antecedents of LMX from the perspective of leaders, concentrating on one specific type of leadership, such as transformational leadership (Wang et al., 2005), servant leadership (Wu et al., 2013), and ethical leadership (Walumbwa et al., 2011). However, studies that examine the effects of ambidextrous leadership congruence on LMX remain limited. Sagie et al. (2002) asserted that loose–tight leadership is positively related to motivation-related variables. LMX is a motivation-related variable characterized through levels of trust, interaction, support and formal and informal rewards (Dienesch and Liden, 1986). It is therefore necessary to explore the relationship between loose–tight ambidextrous leadership (in)congruence and LMX. In this study, we regard four combinations between loose leadership and tight leadership as antecedents according to their different strengths. We also test the effects of loose–tight ambidextrous leadership congruence and incongruence on LMX, further extending theories on LMX.

## Theoretical Background and Hypotheses

### Ambidextrous Leadership Congruence

Ambidextrous leadership combines two seemingly conflicting leadership styles to create a unified strategy that effectively manages tensions (Zacher and Rosing, 2015; Luo et al., 2018). When discussing the context of China, Xing and Liu (2015) have asserted that ambidexterity in leaders helps motivate quality leader–member relationships, enabling them to solve complicated management issues. Transaction–transformation leadership, opening–closing leadership and loose–tight leadership are concrete forms of ambidextrous leadership and are widely used by studies as a typical model of ambidextrous leadership (Masa’deh et al., 2016; Zacher et al., 2016).

Previous studies have explored ambidextrous leadership from the perspective of power (Clark et al., 2009; Raub and Robert, 2013), using ‘loose–tight leadership’ to study management dynamics in the organization. ‘Loose–tight leadership’ reflects a paradox of autonomy versus control (Zhang Y. et al., 2015). Loose leadership refers to a participative leadership style that enables the sharing of power amongst leaders and followers (Vroom, 1997). In a loose leadership, managers prefer to delegate power and provide autonomy to their employees and promote productivity. Tight leadership refers to the directive leadership style, which uses specific frameworks and actions strictly in line with the thoughts of leaders (Sagie et al., 2002). In tight leadership, managers tend to use disciplines and regulations to manage employee behavior.

**FIGURE 1:**
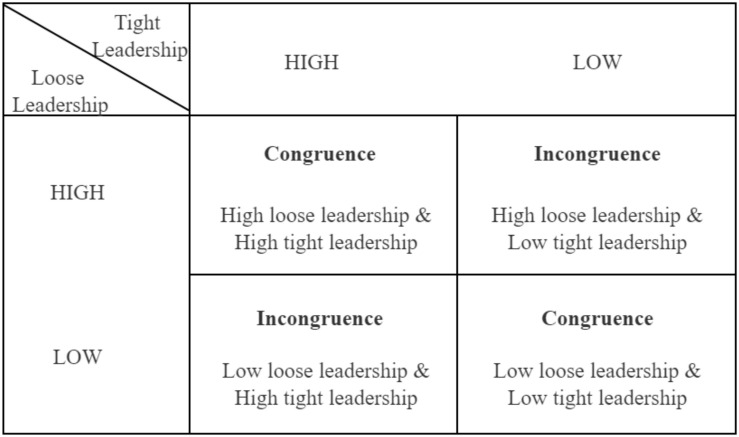
Loose-tight leadership congruence.

Much attention has been paid to loose and tight leadership along with the impact and effectiveness of both leadership styles (Kanungo, 1997). Scholars have observed that leaders tend to perform loose leadership when the achievement of a decision requires member commitments and that leadership is tighter when the information provided to employees is sufficient for decision making (Vroom, 1997). The loose–tight model essentially integrates elements of the traditional participative and directive approaches, indicating that both practices are necessary part of the management process (Shao et al., 2019). However, few studies have explored the combination of loose leadership and tight leadership and their strengths (i.e., high or low). Here, we use four leadership combinations (Figure 1). These are loose–tight ambidextrous leadership congruence, which includes ‘high–high’ and ‘low–low’, and loose–tight ambidextrous leadership incongruence, which includes ‘high–low’ and ‘low–high’.

### Paradox Theory

A paradox occurs when independent elements that seem contradictory coexist in one sphere (Smith and Lewis, 2011). The concept of paradox could be traced back to eastern and western philosophy and psychology, discussed by philosophers including Aristotle, Confucius, Sigmund Freud, and Lao Zi, among others (Schad et al., 2016). Paradoxical theories are also used to describe tensions in the operations and employee dynamics of modern organizations. This theory shows that seemingly opposite ideas can coexist harmoniously and interdependently to form a continuously changing and transforming totality (Cameron and Quinn, 1988). As a sector, the tourist industry embraces innovation, and paradox theory offers insights for hotels, indicating potential ways to manage tensions inside and outside of the organization.

### Loose–Tight Leadership Congruence and LMX

Leader–member exchange (LMX) describes the relationship between leaders and other individuals, emphasizing an effective, mature and reciprocal exchange which benefits all parties (Graen and Uhl-Bien, 1995). This relationship is developed through three stages, ‘role taking’, ‘role making’ and ‘role routinisation’ (Zhang Y. et al., 2015). ‘Role taking’ refers to the process of leaders assigning tasks to individuals and evaluating their responses in initial interactions. ‘Role making’ describes regular communications about ongoing delegations, feedback and role negotiations between leaders and individuals (Dienesch and Liden, 1986). ‘Role routinisation’ refers to the process of formalizing the relationship between leaders and individuals (Graen and Scandura, 1987). These processes foster leader–member relationships through transactional employment contracts and related psychological contracts (Zhang X. et al., 2015).

Ambidextrous leadership has gained attention in previous research on leadership (Zacher and Wilden, 2014) and literature examining paradox theory (Zhang Y. et al., 2015). Loose and tight leadership techniques affect work outcomes of individual workers (Sagie et al., 2002). Using paradox theory, we examine the effects of loose–tight leadership congruence and incongruence on LMX performance.

When leaders use loose leadership and tight leadership simultaneously, they tend to adopt diverse characteristics. We propose that various characteristics facilitate the dyadic interactions, enabling better LMX at every stage of the exchange process. When loose and tight leadership are congruent, leaders do not emphasize one pole in their ‘role taking’ and instead balance both techniques. This counterbalance contributes to a high level of loyalty and commitment from individuals and creates a good relationship between leaders and subordinates (Liu et al., 2014). During ‘role making’ tight leadership enables organizations to ensure discipline and goal consistency between hotel managers and individuals, whereas loose leadership promotes autonomy within organizations under certain institutions. When loose leadership and tight leadership are congruent, individuals perceive, interpret and share the decisions of leaders accurately and strive for work goal creatively.

During ‘role routinisation’, ‘complementarity advantages’ play a role in ambidextrous leadership congruence. Complementary advantages refers to the joint use of participants in shared coordination devices in ways that benefit both parties (Fiske, 2000). It creates a balance between authorisation and directive in the working process. This balance helps the manager avoid making risky decisions and bypasses the perception biases of individuals (Sagie et al., 2002). Therefore, congruence in loose and tight leadership, forms a stable and long-term relationship between leaders and individuals.

In contrast, loose–tight leadership incongruence can be detrimental to LMX. Without loose leadership, excessive tight leadership may attenuate the flexibility of individuals. Without tight leadership, excessive loose leadership may weaken the regulations important to teamwork (Somech, 2006; Martin et al., 2013). Above all, we predict that loose–tight leadership congruence (the congruence of loose leadership and tight leadership) enhances the quality of LMX through role taking, role making and role routine.

Hypothesis 1: Congruence between loose leadership and tight leadership is positively related to LMX quality.

Based on paradox theory, loose leadership and tight leadership can be congruent at either high or low levels (i.e., high–high and low–low). At the congruence of high–high, hotel managers possess the characteristics of loose leadership and tight leadership (Zhang Y. et al., 2015). Employees can have high levels of autonomy; voice; participation in decision making (results of high loose leadership) or a deep level of understanding of organizational goals, work direction and the expectations of leaders (results of high tight leadership). As a result, the motivation of employees to coordinate is strengthened, thereby influencing them to participate widely and devote themselves fully to a specific task. The quality of ‘role taking’, ‘role making’ and ‘role routinisation’ (three ways to enhance LMX) is thus enhanced. At the congruence of low–low, the loose leadership and tight leadership of hotel managers is not apparent. They are neither concerned about the feedback of subordinates nor do they propose tasks clearly and leaders may not be able to handle ‘role taking’, ‘role making’ and ‘role routinisation’ effectively. Hence, LMX quality is aggravated when congruence is low–low than high–high.

Hypothesis 2: LMX quality is higher when loose leadership and tight leadership are congruent at a high level rather than when loose leadership and tight leadership are congruent at a low level.

Differentiating between the two incongruence combinations that exist in both loose leadership and tight leadership is important, namely, the combination of ‘high loose’ leadership and ‘low tigh’t leadership and ‘low loose’ leadership and ‘high tight’ leadership. We propose that the incongruence effect is asymmetrical, meaning that ‘high loose leadership, low tight leadership’ is more detrimental to LMX quality than ‘low loose leadership, high tight leadership’. Specifically, when incongruence is present in ambidextrous leadership, loose leadership and tight leadership serves as a primary status, and the other is complementary. In ‘high loose leadership, low tight leadership’, hotel leaders show loose leadership characteristics, empowering employees with authority, an effective interaction through which power is shared amongst leaders and followers (Sagie et al., 2002). Followers identify with leaders who emphasize empowerment. These leaders are associated with high quality LMX relationships that are based on respect, trust, and mutual liking (Brown and Treviño, 2006; Hassan et al., 2013). In contrast, at the level of ‘high tight leadership, low loose leadership’, leaders show considerable tight leadership characteristics, and they emphasize discipline and obedience (Sagie et al., 2002). Directive leaders prefer commands, assigned goals and punishments (Sims and Manz, 1996). They endorse theory X management style, which is based on the assumption that employees are not naturally willing to work. This approach leads to ineffective communication between leaders and their subordinates (Liu et al., 2003; Jia et al., 2020). Although the emergence of low loose leadership can partly neutralize the rigidity of tight leadership, the strengths of loose leaderships are insufficient in attenuating the side-effects of tight leadership. Therefore, incongruence between high tight leadership and low loose leadership does not enhance LMX quality more than a combination of approaches.

Hypothesis 3: LMX quality is higher in the combination of ‘high loose leadership, low tight leadership’ than ‘high tight leadership, low loose leadership’.

### Mediation Effect of LMX Between Congruence of Ambidextrous Leadership and Work Outcomes

Individual work outcome is a multidimensional construct (e.g., performance, OCB, satisfaction and creativity). For the purpose of this study, we build on research suggesting that job performance and creativity are distinct criteria that ensure task completion and the flexibility of employees. The positive job performance of employees contributes to organizational goal accomplishment, and creativity contributes to organizational innovation (Hon et al., 2013; Akgunduz, 2015). We focus on the indirect effect of loose–tight ambidextrous leadership on employee work outcomes via LMX and aim to address how and through what mechanisms ambidextrous leadership congruence may influence employee work outcomes.

We have previously hypothesized that (in)congruence in loose leadership and tight leadership is positively associated with LMX. We further postulate that LMX influences the job performance and creativity of employees. Leaders must conduct performance evaluation and resource allocation, meaning they are able to provide expanded resources and strong support to employees. In this situation, ‘role taking’, ‘role making’ and ‘role routinisation’ establish a high-quality LMX relationship (Zhang Y. et al., 2015). Prior studies have stated and empirically shown positive relationships between LMX and individual work outcomes, such as job performance and creativity (Park et al., 2015; Aleksić et al., 2017).

**FIGURE 2:**
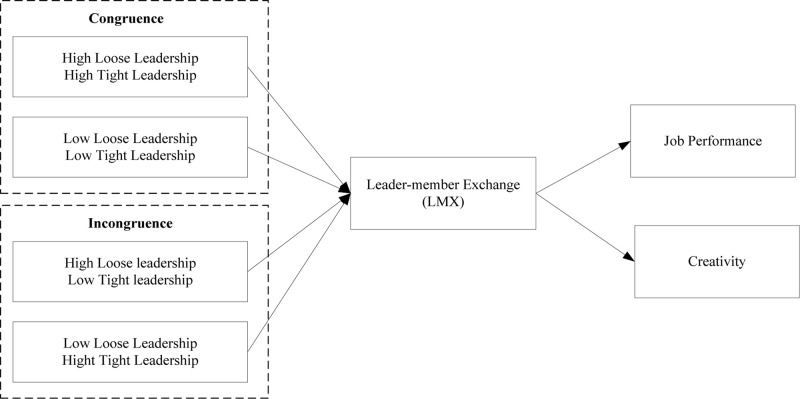
Conceptual model of this research.

Role theory provides additional evidence for the relationship between ambidextrous leadership and employee work outcomes. The central idea in role theory is that people are socialized or conditioned to play various roles that help maintain a stable society or social order (Kahn et al., 1964). This suggests that leaders have diverse roles and tackle different relational issues. Ambidextrous leadership provides organization regulations and flexibility simultaneously. When loose leadership and tight leadership are congruent, there is a better quality of LMX than when incongruence is used. In this situation, employees know what to do and think independently about how to do it. Therefore, employees easily attain goals in organizations and achieve better job performance. Moreover, when loose leadership and tight leadership are congruent diversified leadership roles provide a harmonious working atmosphere, which contributes to the creativity of employees. Conceptual model of this research is shown in Figure 2.

Hypothesis 4.1: LMX quality mediates the relationship between ambidextrous leadership congruence and employee performance.

Hypothesis 4.2: LMX quality mediates the relationship between ambidextrous leadership congruence and employee creativity.

## Methodology

We posit that these two seemingly conflicting leadership styles can coexist with different strengths, contributing to leadership fit-related phenomena and enriching understanding of the ramifications of ambidextrous leadership (Zhang Y. et al., 2015). More research is required in order to explain the mechanisms of ambidextrous leadership and it is important to identify such mechanisms using quantitative research (Jia et al., 2018), specifically polynomial regression and surface analysis, which contribute to testing different relationships with congruence-related issues (Edwards and Parry, 1993; Edwards and Cable, 2009). Thus, we adopt polynomial regression and surface analysis to investigate the inner mechanisms of ambidextrous leadership congruence or incongruence.

### Samples

We conducted a two-wave survey to test the hypotheses. Firstly, we obtained approval to conduct the survey from a top executive of a company in China, with 1,400 chain hotel sub-branches and more than 59,000 employees in the hospitality industry. Secondly, the human resource department of the company randomly generated a list of 550 full-time employees who could participate. These employees worked at 30 sub-branches in Shanghai, with nearly 40 employees in every sub-branch. We informed all the identified participants that the survey was purely for academic research and that their responses would remain confidential. We indicated that participants who completed the two-wave questionnaire would receive 30 Yuan (approximately US$5) for their time. We followed an established back-translation procedure to convert the original survey to the local language (Brislin, 1970). To ensure the confidentiality of survey responses, we instructed respondents to return the answered questionnaires in sealed envelopes.

We adopted a time-lagged design to minimize common method variance (Podsakoff et al., 2003). At Time 1 (T1), employees received their first survey, which assessed loose leadership, tight leadership and control variables. At Time 2 (T2, two months later), we asked the participants who completed the first survey to sequentially rate LMX job performance and creativity. This temporal separation helped alleviate common method bias and strengthen the inference of directionality in the relationships between loose–tight leadership, LMX, job performance and creativity. A total of 400 employees completed the first wave survey, and 321 of 400 employees completed the second wave survey. After matching the data from the two periods and deleting missing data, the final dataset included 301 respondents.

The response rate was 75.3%. Amongst all respondents, 41.7% were male. The average age of respondents was 28.32 years old (SD = 1.58). Their educational background included junior college (27.6%), bachelor’s degree (58.8%) and master’s degree (13.6%). Of the 30 sub-branches invited to participate, the average amount of years during which they had been operating was 14.28 (SD = 0.78). With regard to the size of sub-branches, 18.9% of respondents were from hotels with fewer than 10 employees, 43.5% from firms with 11 to 20 employees, 23.6% from firms with 21 to 30 employees and 14.0% from firms with 31 to 40 employees.

### Measures

#### Loose Leadership

We used the three-item scale developed by Sagie et al. (2002) to measure loose leadership. We instructed the participants to indicate the extent to which they agreed or disagreed with the statements, including ‘My supervisor solves problems in my department’ and ‘My supervisor initiates changes in my department’ (1 = strongly disagree, 5 = strongly agree). The Cronbach’s alpha for this scale was 0.83.

#### Tight Leadership

We used the six-item scale developed by Sagie et al. (2002) to measure tight leadership. We instructed the participants to indicate the extent to which they agreed or disagreed with the statements, including ‘My supervisor is inspirational, able to motivate by articulating effectively the importance of the task’ and ‘My supervisor provides inspiring strategic and organizational goals’ (1 = strongly disagree, 5 = strongly agree). The Cronbach’s alpha for this scale was 0.73.

#### LMX

We used the seven-item scale developed by Wang et al. (2005) to measure LMX. We instructed the participants to indicate the extent to which they agreed or disagreed with the statements, including ‘My supervisor is inclined to use his or her power to help me solve problems in my work’ and ‘My supervisor recognises my potential’ (1 = strongly disagree, 5 = strongly agree). The Cronbach’s alpha for this scale was 0.88.

#### Job Performance

We used the four-item scale developed by Farh et al. (1991) to measure job performance. We instructed the participants to indicate the extent to which they agreed or disagreed with statements, including ‘I am one of the best employees that we have working for us’ and ‘I am one of the most productive employees’ (1 = strongly disagree, 5 = strongly agree). The Cronbach’s alpha for this scale was 0.71.

#### Creativity

We used the four-item scale developed by Farmer et al. (2003) to measure creativity. We instructed the participants to indicate the extent to which they agreed or disagreed with the statements, including ‘I seek new ideas and ways to solve problems’ and ‘I generate ground breaking ideas related to the field’ (1 = strongly disagree, 5 = strongly agree). The Cronbach’s alpha for this scale was 0.74.

#### Control Variables

Prior research suggests that LMX quality may be related to the demographic of employees and organizational similarity (e.g., Bauer and Green, 1996). We chose the demographic characteristics of employees (i.e., age, gender, and years of education) and hotel characteristics (i.e., hotel scale and hotel age) as control variables. These variables were likely associated with the job performance and creativity of employees.

### Procedure and Data Analysis

We used polynomial regression and surface analysis method to test our hypothesis via SPSS 22.0 (Edwards and Parry, 1993) in the different influences of ambidextrous leadership combination. Hypotheses 1, 2 and 3 are estimated by the following equation:

$$\mathrm{LMX}=a_{0}+a_{1}\,\left(L\,L\right)+a_{2}\,\left(T\,L\right)+a_{3}\,\left(L\,L^{2}\right)+a_{4}\,\left(L\,L\times T\,L\right)+a_{5}\,\left(T\,L^{2}\right)+e,$$

where *LMX* is the dependent variable. *LL* represents loose leadership, and *TL* represents tight leadership. We created three new variables by calculating *LL* and *TL*. *LL*^2^ represents the square of *LL*. *TL*^2^ represents the square of *TL*. *LL-TL* represents *LL* multiplied by *TL*. *e* represents error. Then, we used the regression coefficients to plot the three-dimensional response surfaces in which *LL* and *TL* were plotted on the perpendicular horizontal axes, and *LMX* was plotted on the vertical axis (Edwards and Parry, 1993).

With regard to Hypotheses 4.1 and 4.2, we combined the five polynomial terms (*LL, TL, LL^2^, TL^2^* and *LL-TL*) into a block variable to examine the joint effect. The respective weights are the estimated regression coefficients in the polynomial regression. The indirect effect of ambidextrous leadership congruence or incongruence on work outcome via *LMX* can be calculated as a product of the coefficient of the block variable on *LMX*. The coefficient of *LMX* predicting the outcome variable when the direct effect of ambidextrous leadership is included in the regression.

## Results

### Measurement Model Results

We analyzed the collected data using SEM with AMOS 6.08 to test the distinctness of variables. The measurement model consists of items for five latent variables (loose leadership, tight leadership, LMX, job performance and creativity). Table 1 shows the results of model fit comparisons. The hypothesized five-factor model shows satisfactory fit (χ^2^/df = 3.10, CFI = 0.92, TLI = 0.91, AGFI = 0.90, RMSEA = 0.08) and has significantly better fit-indices than all of alternative models. Thus, our measurement instruments have desirable discriminant validity.

**TABLE 1:** Confirmatory factor analyses of measurement models.

**Model**	**χ^2^/df**	**CFI**	**TLI**	**AGFI**	**RMSEA**
One-factor model (LL+TL+LMX+JP+CC)	14.50	0.44	0.35	0.34	0.27
Two-factor model	11.06	0.58	0.51	0.50	0.24
(LL+TL; LMX+JP+CC)					
Three-factor model	9.73	0.77	0.71	0.70	0.13
(LL+TL; LMX; JP+CC)					
Four-factor model	5.03	0.85	0.81	0.80	0.10
(LL; TL; LMX; JP+CC)					
Five-factor model	3.10	0.92	0.91	0.90	0.08
(LL; TL; LMX; JP; CC)					

*LL, loose leadership; TL, tight leadership; LMX, leader-member exchange; JP, job performance; CC, creativity.*

### Descriptive Statistics and Correlations

Table 2 shows the means, standard deviations and intercorrelations of these variables. As proposed, LMX is positively related to loose leadership (*r* = 0.36, *p* < 0.01) and tight leadership (*r* = 0.47, *p* < 0.01). Job performance (*r* = 0.46, *p* < 0.01) and creativity (*r* = 0.54, *p* < 0.01) are positively related to LMX.

**TABLE 2:** Means, standard deviations, and intercorrelations.

**Variables**	**MEAN**	**SD**	**1**	**2**	**3**	**4**	**5**
Loose leadership	3.89	0.66	(0.83)				
Tight leadership	3.94	0.48	0.55^∗∗^	(0.73)			
LMX	3.80	0.58	0.36^∗∗^	0.47^∗∗^	(0.88)		
Job performance	4.01	0.60	0.16^∗∗^	0.38^∗∗^	0.46^∗∗^	(0.71)	
Creativity	4.02	0.47	0.16^∗∗^	0.23^∗∗^	0.54^∗∗^	0.53^∗∗^	(0.74)

**p < 0.01, *p < 0.05.

### Main Effects

Table 3 shows the possibility of a second-order polynomial effect (Edwards and Parry, 1993) because of the significant increase in *R*^2^ (△*R*^2^ = 0.01, *p* < 0.001). Thus, a nonlinear relationship between the effect of loose–tight ambidextrous leadership congruence/incongruence and LMX exists.

**TABLE 3:** Polynomial regression results for LMX.

**Variables**	**LMX**		
	**M1**	**M2**	**M3**
Sex	0.05	0.078	0.055
Age	–0.14	–0.149	−0.138*
Education background	0.14**	0.137**	0.145***
Hotel Age	–0.02	–0.022	–0.015
Hotel Scale	0.22***	0.137	0.139***
LL		−0.090**	−0.068*
TL		0.403***	0.369***
LL^2^			0.012
LL × TL			0.075*
TL^2^			−0.074***
R^2^	0.26	0.60***	0.611***
△R^2^		0.336***	0.014***
LL = TL			
Slope_1_			0.301***
Curvature_1_			0.012
LL = −TL			
Slope_2_			−0.437***
Curvature_2_			−0.137*

LL, loose leadership; TL, tight leadership. ***p < 0.001; **p < 0.01; *p < 0.05.

As also shown in Table 3, the surface curvature along the incongruence line (TS = −TF) was negative (*M3*, *Curvature*_2_ = −0.14, *p* < 0.05), indicating the LMX quality increased significantly when loose leadership and tight leadership were congruent. Thus, Hypothesis 1 is supported.

The slope of the surface along the congruence line (LL = TL) was significantly positive (*M3*, *Slope*_1_ = 0.30, *p* < 0.001), and the curvature of surface along the congruence line (LL = TL) was not significant (*M3*, *Curvanture*_1_ = 0.01, *n.s.*), indicating that significant difference existed. Thus, Hypothesis 2 is supported.

**FIGURE 3:**
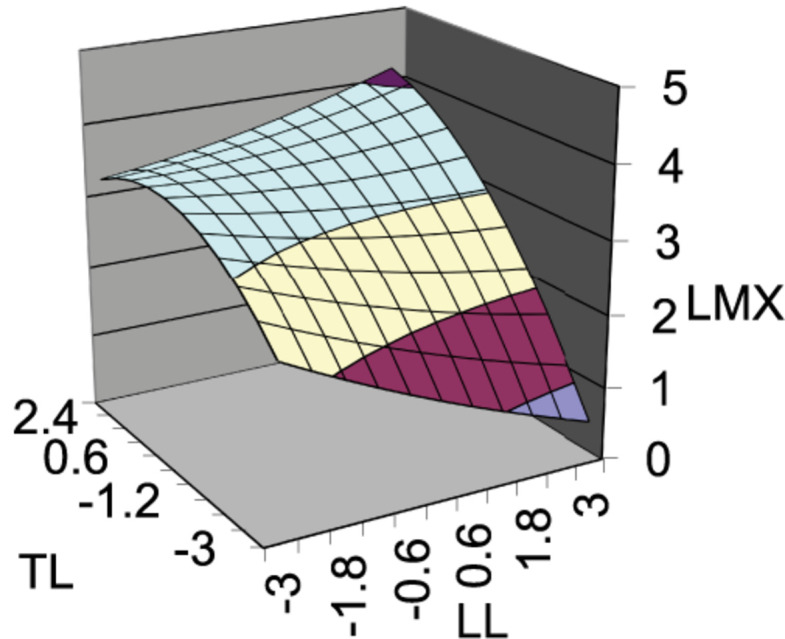
Response surface.

The slope of surface along the incongruence line (LL = −TL) was significantly negative (*M3*, *Slope*_2_ = −0.44, *p* < 0.001). Furthermore, as calculated, the lateral shift quantity was -1.60, indicating that LMX quality increased significantly when tight leadership was higher than loose leadership. Therefore, Hypothesis 3 is not supported. To interpret these results holistically, we plotted the overall response surface by using the coefficient estimates (see Figure 3).

### Mediation Effect of LMX

To test the mediation effect of LMX between ambidextrous leadership congruence/incongruence, we estimated bias-corrected/percentile confidence intervals by using bootstrapping method to test the mediation effect of the block variable (Johnson, 2001; Cole et al., 2013). If bias-corrected/percentile bootstrapped CIs of the indirect effect exclude zero, the mediation effect of LMX is significant. As shown in Tables 4, 5, the bias-corrected bootstrapped CIs of the indirect effect of ambidextrous leadership congruence/incongruence on job performance (95% CI [0.34, 0.71]) and creativity (95% CI [0.65, 0.99]) are significant when LMX is considered (a pattern consistent with partial mediation). Thus, Hypotheses 4.1 and 4.2 are supported.

**TABLE 4:** Indirect effects of ambidextrous leadership on job performance via LMX.

**Variable**	**Estimate**	**Product of Coefficients**		**Bootstrapping**			
				**Bia-Corrected 95%CI**		**Percentile 95%CI**	
		**SE**	**Z**	**Lower**	**Upper**	**Lower**	**Upper**
**Total Effect**							
Block? Performance	0.468	0.080	5.838	0.328	0.640	0.295	0.607
**Indirect Effect**							
Block? Performance	0.520	0.095	5.474	0.338	0.714	0.334	0.712
**Direct Effect**							
Block? Performance	−0.052	0.086	−0.604	0.192	0.135	−0.235	0.106

**TABLE 5:** Indirect effects of ambidextrous leadership on creativity via LMX.

**Variable**	**Estimate**	**Product of Coefficients**		**Bootstrapping**			
				**Bia-Corrected 95%CI**		**Percentile 95%CI**	
		**SE**	**Z**	**Lower**	**Upper**	**Lower**	**Upper**
**Total Effect**							
Block?Creativity	0.693	0.095	7.295	0.517	0.884	0.515	0.881
**Indirect Effect**							
Block? Creativity	0.826	0.088	9.386	0.651	0.994	0.647	0.989
**Direct Effect**							
Block? Creativity	−0.133	0.086	−1.547	−0.301	0.037	−0.295	0.045

## Discussion

This study adopted a paradox theory framework to examine the extent to which ambidextrous leadership congruence and incongruence influence LMX quality with regard to the job performance and creativity of employees. First, we found that ambidextrous leadership leads to better LMX quality when the strengths of loose leadership and tight leadership are congruent rather than incongruent. Second, high loose–high tight leadership can enhance LMX quality, compared with low loose–low tight leadership. Third, LMX quality mediates the relationship between ambidextrous leadership and the job performance and creativity of employees.

This result shows incongruence between high tight leadership and low loose leadership enhanced LMX quality better than incongruence between high loose leadership and low tight leadership, which may be opposite to Hypothesis 3. This result may be because high tight leadership and low loose leadership mainly show directive characteristics (low participative characteristics as complementary). Accordingly, supervisors control decision-making authority and job-related resource assignment. According to LMX theory, leaders are not able to treat all employees equally (Dienesch and Liden, 1986; Wu et al., 2013). Employees are eager to construct effective, mature and reciprocal exchange relationships with supervisors to obtain greater resources.

### Theoretical Implications

This study makes several contributions to literature on leadership and hospitality management. First, our study has extended paradoxical leadership theory and developed and examined the ambidextrous leadership model of hotel managers. As mentioned, prior leadership studies either emphasized one pole of leadership (e.g., Clark et al., 2009; Wu et al., 2013) or only interpreted the legitimacy and theoretical basis of the coexistence of ambidextrous leadership (e.g., Zhang Y. et al., 2015). However, these studies overlooked situations when two poles of ambidextrous leadership coexist. Our study provides a new perspective on ambidextrous leadership in that we attempt to interpret how loose leadership and tight leadership coexist in one situation. We construct four ambidextrous leadership combinations according to the strengths of each leadership (i.e., high or low), namely, high loose–high tight, low loose–low tight, low loose–high tight and high loose–low tight. This study thus contributes to literature on ambidextrous leadership by considering congruence or incongruence between two seemingly conflicting leadership styles.

This study provides a comprehensive overview of how ambidextrous leadership influences employee work outcomes via LMX. We found that LMX mediates the dynamic relationship between ambidextrous leadership congruence/incongruence and the job performance and creativity of hotel employees. Previous studies mainly investigated how a certain leadership style affects LMX quality (Newman et al., 2017; Ospina, 2017), but they did not examine the effect of combinations of these two leadership behaviors on LMX. LMX may best characterize the social exchange dynamics between ambidextrous leaders and followers. Our results show that leadership style is not only exclusive to the labels of the supervisor but also to dynamic characteristics. For example, one supervisor may have different, even opposite, characteristics with different strengths. LMX quality has been developed with the joint cooperation or competition of two leadership styles, that is termed ambidextrous leadership dynamic combination. Ambidextrous leadership leads to a stable condition of two leadership styles and obtains advantages from both styles. Thus, our study provides a dynamic perspective to investigate leader–follower-related issues.

Third, this study demonstrates that loose–tight ambidextrous leadership still caters to the ‘optimum matching principle’, that is effect of congruence is better than incongruence (Yang et al., 2017). Dyadic congruence at high versus low levels of ambidextrous leadership leads to differentiated LMX quality. Our study shows that high loose leadership and high tight leadership are not the contrary when they develop an ambidextrous leadership combination. Accordingly, supervisors possess characteristics of loose leadership and tight leadership (Zhang Y. et al., 2015). Employees may have high levels of autonomy; voice; participation in decision making or a deep level of understanding of organizational goals, work direction and expectation of leaders. Loose leadership and tight leadership can compensate for the disadvantages of one another. Thus, our study provides the theoretical foundation of the effectiveness of ambidextrous leadership.

### Managerial Implications

In addition to theoretical implications, our findings offer several practical implications. In today’s fast-paced environment, competing pressures force hotel managers to change from ‘either–or’ leadership strategy into ‘both–and’ leadership strategy. Firstly, ambidextrous leadership contributes to dealing with complicated problems with increasing uncertainty. When managing employee behavior, hotel managers need to facilitate a proactive approach to work, encouraging staff to use autonomy whilst also motivating and disciplining employees. Thus, ambidextrous leadership is a helpful strategy for integrating paradoxes and promoting work outcomes. Secondly, high loose leadership in the hospitality industry motivates employees to improve their self-management skills. High tight leadership contributes to better task completion and job performance. High–high congruence also corresponds well with ancient Chinese Yin–Yang philosophy. Additionally, timely communication between hotel managers and employees is essential, ensuring hotel managers are able to establish high levels of trust, interaction, support, along with formal and informal rewards for employees.

## Limitations and Future Research

This study has several limitations that indicate directions for future research. First, we only choose the size and age of the hotel as control variables at the organizational level, considering the fixed nature of the enterprise (China’s economical chain hotels are mainly private enterprises) and industry. Future research might consider other variables in hotel characteristics related to LMX and industry-specific employee work outcomes, such as the star rating of hotels and the demographic characteristics of hotel managers (Wu et al., 2013).

Second, we measured all the concepts from the perspective of subordinates rather than supervisors. Although we performed the data collection process in two waves, the assessment of certain concepts (e.g., job performance and creativity) from the perspective of two different sources (members and supervisors) might be more objective than results gathered through self-assessment.

Third, our results are specific to the Chinese context. For example, what is regarded as loose leadership or tight leadership in western countries may not necessarily be the same as that in China. In addition, the optimal ambidextrous leadership congruence to motivate employee work outcomes via LMX may also vary. Future studies should compare the impacts of ambidextrous leadership between western and Chinese samples and set culture as control variables on LMX and employee work outcomes.

## Conclusion

Exploring the use of loose and tight leadership techniques in chain hotels in China, this study has discussed how the model of paradox theory connects ambidextrous leadership congruence or incongruence with employee performance and creativity. More specifically, it has discussed how ambidextrous leadership congruence leads to higher LMX quality. When tight leadership and loose leadership are congruent, ‘high loose–high tight’ is more desirable than ‘low loose–low tight’. By contrast, when tight and loose leadership are incongruent, ‘low loose–high tight’ is more desirable than ‘high loose–low tight’ in enhancing LMX quality. Ultimately, LMX quality mediates the relationship between ambidextrous leadership congruence/incongruence and employee work outcomes.

## Data Availability Statement

The datasets generated for this study are available on request to the corresponding author.

## Ethics Statement

The study was approved by the Ethics Committee of the Department of Economics and Management, Tongji University, Shanghai, China. All participants provided written informed consent.

## Author Contributions

ZG and JY conceived and designed the study. ZG and XW designed the questionnaire and collected data. ZG and JY conducted data analysis. ZG, JY, and XW wrote the manuscript. JZ reviewed and edited the manuscript. All authors read and approved the manuscript.

## Conflict of Interest

The authors declare that the research was conducted in the absence of any commercial or financial relationships that could be construed as a potential conflict of interest.
